# Suicide risk following a new cancer diagnosis among Veterans in Veterans Health Administration care

**DOI:** 10.1002/cam4.5146

**Published:** 2022-08-27

**Authors:** Kallisse R. Dent, Benjamin R. Szymanski, Michael J. Kelley, Ira R. Katz, John F. McCarthy

**Affiliations:** ^1^ Veterans Affairs (VA) Serious Mental Illness Treatment Resource and Evaluation Center Office of Mental Health and Suicide Prevention Ann Arbor Michigan USA; ^2^ Veterans Affairs (VA) National Oncology Program Specialty Care Services, VA Washington District of Columbia USA; ^3^ Duke Cancer Institute Durham North Carolina USA; ^4^ Hematology‐Oncology Durham VA Health Care System Durham North Carolina USA; ^5^ VA Office of Mental Health and Suicide Prevention Washington District of Columbia USA

**Keywords:** cancer survivors, suicide, veterans

## Abstract

**Background:**

Cancer diagnoses are associated with an increased risk for suicide. The aim of this study was to evaluate this association among Veterans receiving Veterans Health Administration (VHA) care, a population that has an especially high suicide risk.

**Methods:**

Among 4,926,373 Veterans with VHA use in 2011 and in 2012 or 2013, and without VHA cancer diagnoses in 2011, we assessed suicide risk following incident cancer diagnoses. Risk time was from initial VHA use in 2012–2013 to 12/31/2018 or death, whichever came first. Cox proportional hazards regression models evaluated associations between new cancer diagnoses and suicide risk, adjusting for age, sex, VHA regional network, and mental health comorbidities. Suicide rates were calculated among Veterans with new cancer diagnoses through 84 months following diagnosis.

**Results:**

A new cancer diagnosis corresponded to a 47% higher suicide risk (Adjusted Hazard Ratio [aHR] = 1.47, 95% CI: 1.33–1.63). The cancer subtype associated with the highest suicide risk was esophageal cancer (aHR = 6.01, 95% CI: 3.73–9.68), and other significant subtypes included head and neck (aHR = 3.55, 95% CI: 2.74–4.62) and lung cancer (aHR = 2.35, 95% CI: 1.85–3.00). Cancer stages 3 (aHR = 2.36, 95% CI: 1.80–3.11) and 4 (aHR = 3.53, 95% CI: 2.81–4.43) at diagnosis were positively associated with suicide risk. Suicide rates were highest within 3 months following diagnosis and remained elevated in the 3–6‐ and 6–12‐month periods following diagnosis.

**Conclusion:**

Among Veteran VHA users, suicide risk was elevated following new cancer diagnoses. Risk was particularly high in the first 3 months. Additional screening and suicide prevention efforts may be warranted for VHA Veterans newly diagnosed with cancer.

## Graphical abstract

Veterans with a new cancer diagnosis are at an elevated risk for suicide, with the highest risk in the first 3 months following diagnosis. Additional screening and suicide prevention efforts may be warranted for VHA Veterans newly diagnosed with cancer, especially among high‐risk subtypes including esophageal, head and neck, and lung cancer and cancers diagnosed at stages 3 and 4.

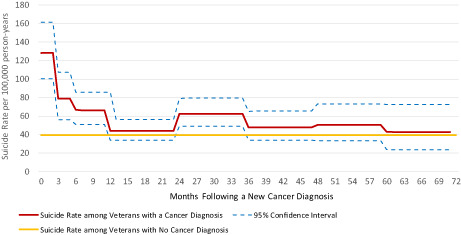

## 1 INTRODUCTION

In 2019, the adjusted suicide rate among Veterans was 52.3% greater than among non‐Veteran US adults.^1^ Among Veterans, those with recent Veterans Health Administration (VHA) encounters had greater suicide risk than other Veterans.^2^ Suicide has many causes and as part of ongoing Veterans Affairs (VA) suicide surveillance it is important to assess potential suicide risk factors.

Recent studies suggest that new cancer diagnoses are positively associated with suicide risk, particularly in the months immediately following diagnosis.^3^, ^4^, ^5^, ^6^, ^7^ Suicide risk corresponding to a cancer diagnosis varies by cancer stage, type, prognosis, and other patient characteristics.^3^, ^4^, ^5^, ^7^ Cancer patients within the US from 1973–2014 had 4.44 times the suicide risk of the general population. This risk was higher immediately following diagnosis, among younger patients, and in more recent years.^3^ Among US adults diagnosed with cancer 2000–2014, odds of suicide were highest in the second month following diagnosis and for distantly metastasized, pancreatic, and lung cancer subtypes.^4^ Furthermore, the suicide standardized mortality ratios corresponding to new cancer diagnoses increased in more recent years, rising from 1.27 in 2000–2007 to 1.58 in 2007–2013.^7^

The Interpersonal Theory of Suicide posits that active suicidal desire is driven by the presence of thwarted belongingness, perceived burdensomeness, and hopelessness that these interpersonal struggles will improve, while the capacity for suicidal behavior is an independent process driven by fearlessness and familiarity with pain.^8^ A cancer diagnosis may represent a life event that triggers both interpersonal struggles, offers little hope for change in the future, and increases a patient's capability for suicide through an increase in pain tolerance and acceptance of death.^8^ Cancer diagnosis and subsequent treatment may diminish social engagement, reciprocal relationships, and perceived belonging, while the physical and financial strain of cancer may increase feelings of burdensomeness. Among cancer patients, physical stressors associated with suicidal behavior include pain, disfigurement, body‐image concerns, and dysfunction.^9^, ^10^, ^11^, ^12^ Financial distress affects approximately 47%–49% of cancer survivors and increases risk for depression and anxiety.^13^, ^14^ Cancer diagnoses can also stress social support networks, leading to relationship troubles.^15^, ^16^ Among Veterans diagnosed with cancer, those reporting relationship problems had a five times greater likelihood of suicidal ideation than those without them.^17^ Physiological factors, such as cytokines produced by tumors and glucocorticoids used to treat cancer, may also increase depression and anxiety risk.^17^ A high prevalence of mental health and substance use disorders among cancer patients may also increase suicide risk among cancer patients.^18^, ^19^

On average, the Veteran population is older than the non‐Veteran US adult population and has a higher burden of comorbid health conditions, including cancer.^20^ Veteran VHA users diagnosed with cancer have an elevated prevalence of suicide risk factors, including PTSD symptoms, suicidal ideation, and mental health and substance use disorders.^17^, ^21^, ^22^ Understanding associations between new cancer diagnoses and suicide risk, overall and adjusting for mental health disorders, is important for enhancing Veteran suicide prevention efforts. Although cancer has been associated with adverse mental health outcomes among Veterans, we know of no studies that have assessed the relative suicide risk of Veteran VHA users who receive a new, or incident, cancer diagnosis. Furthermore, prior studies of cancer and suicide in the US population have often lacked adjustment for comorbid mental health conditions.^3^, ^4^, ^7^

The Veterans Health Administration's Suicide Prevention Now plan aims to identify and target strategies to immediately prevent Veteran suicide, including the identification of high‐risk medical diagnoses. In support of this initiative, the aims of these analyses were to: (1) assess the association between new cancer diagnoses and suicide risk among Veterans engaged in VHA care, adjusting for mental health comorbidities, (2) identify cancer subtypes and post‐diagnosis periods of elevated risk for suicide, and (3) determine whether method of suicide among suicide decedents differs for those with versus without an incident cancer diagnosis.

## 2 METHODS

All analyses were conducted as a part of VA's Office of Mental Health and Suicide Prevention operations and quality improvement activities, and institutional review board was not required.

### 2.1 Cohort

The analytic cohort included 4,926,373 Veterans with VHA inpatient or outpatient encounters in 2011, who did not have VHA documented cancer diagnoses in 2011, and who received VHA care in 2012 or 2013. VHA encounters were assessed using electronic health record data in the VHA Corporate Data Warehouse (CDW).

### 2.2 Incident cancer diagnosis

A Veteran's first cancer diagnosis between their first VHA use in 2012–2013 and 12/31/2018 was identified using the CDW‐Oncology Raw Domain, which is used nationally to capture VHA incident cancer diagnoses and has high sensitivity and specificity.^23^ Cancer diagnosis was categorized by sub‐type and stage at diagnosis. There were 26 sub‐types (see Table 1) and cancer stage was coded from 0 (carcinoma in situ) to 4 (cancer has distant metastases), or as unknown, missing, and not applicable.^24^

**TABLE 1:** Veteran VHA users in 2011 with use in 2012–2013: percent with new cancer diagnosis, overall, and by suicide status

Variable	All veteran VHA users		Veteran VHA users with suicide deaths		Other veteran VHA users^a^		*p*‐value^b^
	*N*	% with Cancer Diagnosis, 2012–2018	*N* ^c^	% with Cancer Diagnosis, 2012–2018	*N*	% with Cancer Diagnosis, 2012–2018	
Veteran VHA users in 2011 with use in 2012 or 2013	4,926,373						
Age on date of first VHA use 2012–2013, mean (SD)	4,926,373	62.5 (15.7)	11,505	60.8 (16.8)	4,914,868	62.5 (15.7)	<0.001
Sex, % female	382,263	7.76	409	3.55	381,854	7.77	<0.001
Any cancer	240,346	4.88	393	3.42	239,953	4.88	<0.001
Cancer sub‐type							
Anal cancer^d^	842	0.02	‐	‐	842	0.02	0.276
Bladder cancer	16,170	0.33	26	0.23	16,144	0.33	0.055
Brain and other nervous system cancer	1321	0.03	‐	‐	1320	0.03	0.385
Colon cancer	15,363	0.31	26	0.23	15,337	0.31	0.098
Esophageal cancer	4354	0.09	17	0.15	4337	0.09	0.032
Head and neck cancer	13,149	0.27	57	0.50	13,092	0.27	<0.001
Kidney and other urinary cancer	10,540	0.21	12	0.10	10,528	0.21	0.011
Leukemia	7603	0.15	12	0.10	7591	0.15	0.171
Liver cancer	9699	0.20	14	0.12	9685	0.20	0.069
Lung cancer	40,279	0.82	67	0.58	40,212	0.82	0.005
Lymphoma	6429	0.13	10	0.09	6419	0.13	0.195
Melanoma	15,906	0.32	25	0.22	15,881	0.32	0.046
Myeloma	4330	0.09	10	0.09	4320	0.09	0.972
Other digestive cancers	1978	0.04	‐	‐	1977	0.04	0.101
Other blood cancers	2282	0.05	‐	‐	2279	0.05	0.509
Other cancer	1233	0.03	‐	‐	1231	0.03	0.233
Pancreas cancer	5258	0.11	‐	‐	5254	0.11	0.014
Sarcoma	1642	0.03	‐	‐	1639	0.03	0.203
Skin cancer	9373	0.19	20	0.17	9353	0.19	0.686
Stomach cancer	3058	0.06	‐	‐	3055	0.06	0.134
Thyroid and other endocrine cancers	2653	0.05	‐	‐	2649	0.05	0.543
Unknown cancer	4041	0.08	‐	‐	4039	0.08	0.008
Male‐specific cancers (*N* Veterans = 5,544,110)							
Prostate cancer	57,295	1.26	63	0.57	57,232	1.26	<0.001
Male reproductive cancer	1138	0.03	‐	‐	1133	0.02	0.182
Female‐specific cancers (*N* Veterans = 382,263)							
Breast cancer	2854	0.75	‐	‐	2851	0.75	0.225
Female reproductive cancer	1060	0.28	‐	‐	1059	0.28	0.366
Cancer stage at diagnosis (*N* Veterans = 4,926,373)							
Stage 0	17,599	0.36	22	0.19	17,577	0.36	0.003
Stage I	56,380	1.14	81	0.70	56,299	1.15	<0.001
Stage II	45,903	0.93	72	0.63	45,831	0.93	<0.001
Stage III	22,687	0.46	52	0.45	22,635	0.46	0.892
Stage IV	37,147	0.75	75	0.65	37,072	0.75	0.205
Stage—not applicable	18,890	0.38	29	0.25	18,861	0.38	0.022
Stage—missing	29,686	0.60	30	0.26	29,656	0.60	<0.001
Stage–unknown	12,054	0.24	32	0.28	12,022	0.24	0.467

Acronyms: SD, standard deviation; VHA, Veterans Health Administration.

^a^ Others without a suicide death includes both those who died of a non‐suicide cause between 1/1/2012 and 12/31/2018 and those who were alive as of 1/1/2019.

^b^ Chi‐squared *p*‐value for difference in prevalence of cancer diagnosis across suicide decedent and non‐suicide decedent groups. For those cancer sub‐types with fewer than five suicide decedents 2012–2018 a Fischer's Exact test was used. Differences in age were assessed using the Satterthwaite *t*‐test for unequal variances after an *F* test for equality of variances suggested unequal variances in age across suicide decedent and non‐suicide decedent groups.

^c^ Suicide counts less than 10 are suppressed per CDC reporting guidelines, indicated with a “‐“

^d^ The cohort did not include any Veterans with both an incident anal cancer diagnosis and a suicide death in 2012–2018. As suicide risk estimation was not feasible, anal cancer was excluded from subsequent analyses.

### 2.3 Outcome

Death by suicide was assessed using the VA/Department of Defense (DoD) Mortality Data Repository (MDR), which includes results from comprehensive searches of the CDC's National Death Index. Table S1 lists ICD‐10 cause of death definitions for suicide and methods of suicide, which included death by firearm, poisoning, suffocation/strangulation, and other.

### 2.4 Covariates

Covariates, including age, sex, diagnoses, and suicide attempts, were assessed on the Veteran's first day of VHA use 2012–2013. Veterans Integrated Service Network (VISN) assignment was based on the Veteran's last facility of VHA use in 2011. Mental health, tobacco use disorder, and other substance use disorders were assessed in the year prior using ICD9 and ICD10 diagnostic codes (Table S2). Suicide attempts in the prior year included VHA ICD diagnoses and site‐reported suicide attempts documented in the Suicide Prevention Applications Network or the Suicide Behavior Overdose Report.

### 2.5 Statistical analysis

We evaluated the prevalence of demographic characteristics and incident cancer diagnoses 2012–2018 (by type and stage) among: (1) all Veteran VHA users, (2) those who died by suicide in 2012–2018, and (3) those who did not die by suicide in 2012–2018. Chi‐squared tests evaluated differences by suicide status. For cancer sub‐types having fewer than five suicide deaths in the follow‐up period, the Fischer's exact test was used. The Satterthwaite *T*‐test was used to determine differences in age. An alpha = 0.05 was used to assess statistical significance.

Cox proportional hazards regression, accounting for time‐varying cancer exposure, assessed associations between an incident cancer diagnosis and suicide risk. Unexposed risk time started at the Veteran's first VHA use in 2012 or 2013; exposed risk time started on the cancer diagnosis date. Risk‐time ended at cancer diagnosis date, date of death, or 12/31/2018, whichever came first. The initial model adjusted for age, sex, and VISN. After accounting for multiple comparisons using the Bonferroni method (35 tests conducted; alpha = 0.5/35 = 0.0014), cancer sub‐types significantly associated with suicide risk were also assessed using a model that adjusted for VISN, age, and sex, and suicide attempts and diagnosis of mental health disorder, tobacco use disorder, and substance use disorder in the prior year.

Suicide rates following an incident cancer diagnosis were calculated for the subset of the cohort who had a new cancer diagnosis in 2012–2018. Suicide rates per 100,000 person‐years were assessed for periods following diagnosis: 0–3, 3–6, 6–12, 12–24, 24–36, 36–48, 48–60, 60–72, and 72–84 months. Risk time started on the Veteran's date of cancer diagnosis and ended on the Veteran's date of death or the end of the risk period, which ever came first. The gamma distribution was used to calculate 95% confidence intervals for the suicide rates.

Chi‐squared tests were used to assess differences in suicide method across three groups of suicide decedents, 2012–2018: Veteran VHA patients (1) without cancer diagnoses in 2012–2018, (2) with a cancer diagnosis within the 3 months prior to death, and (3) with a cancer diagnosis more than 3 months prior to death. A chi‐squared test was also used to evaluate whether the firearm method significantly differed across groups.

## 3 RESULTS

### 3.1 Associations between incident cancer diagnosis and suicide risk, by cancer type and stage

Of the 4,926,373 Veterans with VHA use in 2011 and no VHA cancer diagnoses that year, and who received VHA care in 2012–2013, 240,346 (4.88%) received an incident VHA cancer diagnosis in the period 2012–2018 (Table 1). On average, Veterans were followed for 5.99 (standard deviation [SD] = 1.72) years after their first VHA use in 2012–2013 and for 2.65 years ([SD] = 2.07) following an incident cancer diagnosis.

Table 1 presents the distribution of age, sex, and cancer diagnosis by subtype for the study cohort, overall and by suicide status. Descriptive statistics, which did not account for patient risk‐time, found that suicide decedents, compared with those who did not die from suicide, were on average younger (60.8[SD = 16.8] versus 62.5[SD = 15.7], *p*‐value <0.001) and less likely to be female (Table 1, 3.55% versus 7.77%, *p*‐value <0.001). Incident cancer diagnosis was less common among those who died by suicide than those who either died from non‐suicidal causes (including cancer) or survived the follow‐up period (Table 1, 3.42% versus 4.88%, *p*‐value <0.001). Cancer sub‐types for which cancer was more common among suicide decedents included esophageal (*p*‐value = 0.030) and head and neck cancer (*p*‐value <0.0001). Cancer sub‐types for which cancer was less common among suicide decedents included kidney and other urinary (*p*‐value = 0.011), lung (*p*‐value = 0.005), melanoma (*p*‐value = 0.046), pancreatic (*p*‐value = 0.014), unknown (*p*‐value = 0.008), and prostate cancer (*p*‐value <0.001). and stages 0 (*p*‐value = 0.003), 1 (*p*‐value <0.001), 2 (*p*‐value<0.001), and reports of “not applicable” (*p*‐value = 0.022), and “missing” (Table 1, *p*‐value <0.001).

Table 2 presents findings from proportional hazards regression analyses for any new cancer diagnosis overall, for each of the 25 cancer sub‐types with some suicides in the follow‐up period, and for cancer stage at diagnosis. Among 4,926,373 Veteran VHA users, a new cancer diagnosis was positively associated with suicide risk. After adjusting for age, sex, VISN, VHA documented suicide attempt in the prior year, and diagnosis of mental health, tobacco use, and other substance use disorders in the prior year, a new cancer diagnosis corresponded to a 47% (HR = 1.47, 95% CI: 1.33, 1.63) higher suicide risk than for those without a new cancer diagnosis (Table 2).

**TABLE 2:** Suicide hazard ratios corresponding to an incident cancer diagnosis, by type and stage

Cancer Subtype^a^	Model 1: Adjusting for VISN, age, and sex		Model 2^b^: Adjusting for VISN, Age, Sex, and mental, tobacco use, and other substance use disorder and suicide attempt in the prior year	
	HR (95% confidence interval)	*p*‐value^c^	HR (95% confidence interval)	*p*‐value
Any cancer	1.56 (1.41, 1.72)	**<0.001**	1.47 (1.33, 1.63)	**<0.001**
Bladder cancer	1.38 (0.94, 2.03)	0.103	N/A	
Brain & other nervous system cancer	1.34 (0.19, 9.53)	0.770	N/A	
Colon cancer	1.48 (1.01, 2.17)	0.047	N/A	
Esophageal cancer	6.53 (4.05, 10.51)	**<0.001**	6.01 (3.73, 9.68)	**<0.001**
Head & neck cancer	3.97 (3.06, 5.15)	**<0.001**	3.55 (2.74, 4.62)	**<0.001**
Kidney & other urinary cancer	0.94 (0.54, 1.66)	0.835	N/A	
Leukemia	1.47 (0.83, 2.59)	0.183	N/A	
Liver cancer	2.03 (1.20, 3.43)	0.008	N/A	
Lung cancer	2.69 (2.12, 3.43)	**<0.001**	2.35 (1.85, 3.00)	**<0.001**
Lymphoma	1.43 (0.77, 2.66)	0.258	N/A	
Melanoma	1.20 (0.81, 1.77)	0.372	N/A	
Myeloma	2.30 (1.24, 4.26)	0.008	N/A	
Other digestive cancer	0.62 (0.09, 4.40)	0.633	N/A	
Other blood cancers	1.26 (0.41, 3.90)	0.689	N/A	
Other cancer	1.96 (0.49, 7.82)	0.343	N/A	
Pancreas cancer	1.96 (0.74, 5.23)	0.177	N/A	
Sarcoma	1.83 (0.59, 5.67)	0.296	N/A	
Skin cancer	1.70 (1.10, 2.64)	0.018	N/A	
Stomach cancer	1.36 (0.44, 4.21)	0.599	N/A	
Thyroid & other endocrine cancers	1.17 (0.44, 3.13)	0.750	N/A	
Unknown cancer	1.12 (0.28, 4.49)	0.871	N/A	
Male‐specific cancers (*N* Veterans = 4,544,110)				
Prostate cancer	0.78 (0.61, 1.00)	0.051	N/A	
Male reproductive cancer	3.18 (1.32, 7.62)	0.010	N/A	
Female‐specific cancers (*N* Veterans = 382,263)				
Breast cancer	2.04 (0.65, 6.36)	0.221	N/A	
Female reproductive cancer	1.64 (0.23, 11.67)	0.624	N/A	
Cancer stage				
Stage 0	0.92 (0.61, 1.40)	0.705	N/A	
Stage 1	1.12 (0.90, 1.39)	0.317	N/A	
Stage 2	1.21 (0.96, 1.52)	0.114	N/A	
Stage 3	2.52 (1.92, 3.31)	**<0.001**	2.36 (1.80, 3.11)	**<0.001**
Stage 4	3.78 (3.01, 4.74)	**<0.001**	3.53 (2.81, 4.43)	**<0.001**
Stage—NA	1.72 (1.19, 2.47)	0.004	N/A	
Stage—missing	1.17 (0.82, 1.67)	0.394	N/A	
Stage—unknown	2.30 (1.63, 3.26)	**<0.001**	2.19 (1.55, 3.10)	**<0.001**

Acronyms: HR, hazard ratio; VISN, Veterans integrated services network.

^a^ Analyses for anal cancer were excluded because there were zero suicides corresponding to a new anal cancer diagnosis, making hazard ratios uninterpretable.

^b^ Model 2 was only run for cancer sub‐types that had a significant association with suicide (alpha = 0.0014). Cancer sub‐types that did not meet this threshold have an N/A for model 2. *P*‐values below the Bonferroni‐adjusted alpha are bold.

^c^ The Bonferroni method was used to adjust for multiple comparisons. Alpha for statistical significance = 0.0014.

Across 26 cancer subtypes and 8 cancer stages (0–4, missing, NA, and unknown), adjusting for multiple comparisons, the following subtypes had a significant positive association with suicide risk: esophageal, lung, and head and neck cancer, cancers diagnosed at stages 3 and 4, and cancers with an unknown stage at diagnosis (Table 2). Adjusting for mental health and substance use disorder comorbidities at diagnosis attenuated but did not alter the significance or direction of these associations (Table 2). After adjusting for covariates, the cancer sub‐type associated with the highest suicide risk was esophageal cancer (adjusted hazards ratio[aHR] = 6.01, 95% CI: 3.73, 9.68), followed by head and neck (aHR = 3.55, 95% CI: 2.74, 4.62), and lung cancer (Table 2, aHR = 2.35, 95% CI: 1.85, 3.00). The cancer stage with the highest suicide risk was stage 4 (aHR = 3.53, 95% CI: 2.81, 4.43) followed by stage 3 (aHR = 2.36, 95% CI: 1.80, 3.11) and “unknown stage” at diagnosis (Table 2, aHR = 2.19, 95% CI: 1.55, 3.10).

### 3.2 High suicide risk periods following a new cancer diagnosis

**FIGURE 1:**
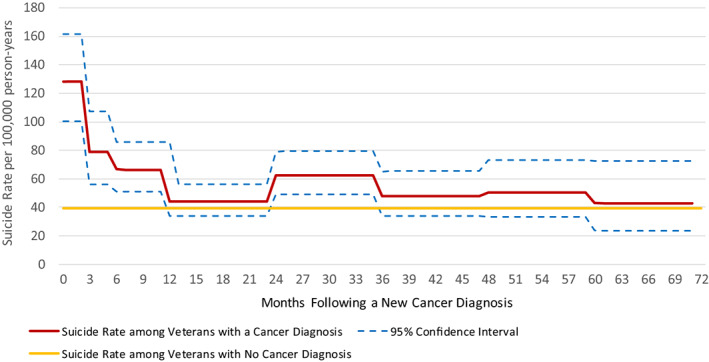
Suicide Rates per 100,000 person‐years following a New Cancer Diagnosis January 1st, 2012–December 31st, 2018 among Veteran VHA Users. Suicide rates are shown only through 72 months as rates for the 72–84 months following cancer diagnosis are suppressed due to suicide counts <10.

Table 3 presents suicide rates per 100,000 person‐years in the months following a new cancer diagnosis. Among 240,410 Veteran VHA users with a new cancer diagnosis 2012–2018, 393 had a subsequent death by suicide in this period. Suicide rates were highest during the first 3 months following diagnosis (rate = 128.3, 95% CI: 100.4, 161.6), accounting for 18.3% of the 393 documented suicides (Table 3 & Figure 1). Relative to those without any cancer diagnosis (rate = 39.4, 95% CI: 38.7, 40.2), rates among cancer patients remained elevated during the 3–6 months (rate = 78.7, 95% CI: 56.2, 107.2) and 6–12 months (rate = 66.6, 95% CI: 50.8, 85.7) following diagnosis (Table 3 and Figure 1).

**TABLE 3:** Suicide rates following any new cancer diagnosis among Veteran VHA users

Time interval following a new cancer diagnosis	*N*, Veterans alive at the beginning of the interval	*N*, suicides in risk period^a^	Suicide rate^a^ per 100,000 person‐years (95% confidence interval)
0–3 months following cancer diagnosis	240,410	72	128.3 (100.4, 161.6)
3–6 months following cancer diagnosis	215,440	40	78.7 (56.2, 107.2)
6–12 months following cancer diagnosis	197,323	60	66.6 (50.8, 85.7)
12–24 months following cancer diagnosis	168,501	65	44.1 (34.1, 56.3)
24–36 months following cancer diagnosis	127,454	70	62.7 (48.9, 79.2)
36–48 months following cancer diagnosis	95,734	39	47.8 (34.0, 65.3)
48–60 months following cancer diagnosis	67,741	28	50.5 (33.5, 73.0)
60–72 months following cancer diagnosis	43,184	*14*	*43.1 (23.6, 72.4)*
72–84 months following cancer diagnosis	21,459	‐	‐

^a^ Suicide rates based on suicide counts less than 10 are suppressed per CDC reporting guidelines, indicated with a “‐.” Italicized rates are based on counts less than 20, which indicate unstable rates.

### 3.3 Method of suicide by presence and proximity of cancer diagnosis

11,505 patients died by suicide between their first VHA use in 2012–2013 and 12/31/2018. Firearms were involved in 70.6% of these deaths (Table 4). Firearm involvement was most common among those who died by suicide within 3 months of a new cancer diagnosis (86.3%) and was more common among those who died by suicide more than 3 months following a cancer diagnosis (81.3%) than those without a VHA‐documented cancer diagnosis prior to death by suicide (70.2%). The use of firearms significantly differed across cancer diagnosis group (Table 4, *p*‐value <0.001).

**TABLE 4:** Suicide method by presence and proximity of new cancer diagnosis on date of death

Suicide method	No cancer diagnosis 2012—date of death		New cancer diagnosis in 3 months prior to date of death		New cancer diagnosis more than 3 month prior to date of death		*p*‐value^a^
	*N*	%	*N* ^b^	%	*N* ^b^	%	
Across all methods	11,112		73		320		<0.001
Firearm	7804	70.2	63	86.3	260	81.3	
Poisoning	1333	12.0	‐	‐	34	10.6	
Strangulation	1348	12.1	‐	‐	17	5.3	
Other	627	5.6	‐	‐	‐	‐	

^a^ Chi‐Squared test indicates that method significantly differed across cancer diagnosis group. Chi‐squared tests specific for the firearm method also showed that use of firearm significantly differenced across group (*p*‐value <0.001).

^b^ Suicide counts less than 10 are suppressed per CDC reporting guidelines, indicated with a “‐”

## 4 DISCUSSION

Cancer was less common among Veteran VHA users who died by suicide when compared with other Veteran VHA users. However, after accounting death by non‐suicidal causes of death and adjusting for important confounders using cox proportional hazards regression, an incident cancer diagnosis was associated with increased suicide risk. Cancer sub‐types with increased suicide risk included esophageal, head and neck, and lung cancer. Cancer stages at diagnosis associated with increased suicide risk included stages 3, 4, and unknown. Suicide risk was particularly high in the first 3 months following a new cancer diagnosis, and in this period, deaths were more likely to involve firearms.

Study findings are consistent with the literature indicating positive associations between an incident cancer diagnosis and suicide risk among the general US adult population, with risk highest in the months immediately following diagnosis.^3^, ^4^, ^7^, ^25^ As with this study, prior work has shown that individuals diagnosed at more advanced stages^3^, ^4^, ^5^, ^6^, ^7^ and with lung,^3^, ^4^, ^5^ esophageal^5^, ^6^, ^7^ or head and neck^3^, ^9^, ^26^ cancer were at elevated risk for suicide. There are several factors which may increase suicide risk within these cancer subtypes.^9^, ^10^, ^11^, ^12^ ^,^ First, disruptions to basic functions such as swallowing, talking, and breathing may contribute to reduced quality of life which is associated with higher risk of suicidal behavior in cancer patients.^10^, ^11^ Second, the poor prognosis corresponding to these subtypes likely leads to feelings of hopelessness.^10^, ^11^ Third, due to lung cancer's associations with well‐known, modifiable behaviors there may be a unique component of self‐blame which contributes to poorer psychological adjustment and subsequent elevations in suicide risk.^27^

In contrast to the current literature, we did not find evidence for an increase in suicide risk (after adjusting for multiple comparisons) corresponding to colorectal,^3^, ^4^, ^6^ pancreatic,^4^, ^5^, ^7^ prostate,^3^ bladder,^3^ gastric,^28^ or leukemia cancers.^3^ Limited power within these cancer types may account for some of these observed differences. For cancer sub‐types with sufficient power, such as prostate cancer, our findings may also represent differences specific to the Veteran VHA user population.

Among Veteran suicide decedents, those with a cancer diagnosis used firearms more often than those without cancer and were most frequently involved among suicide decedents with a cancer diagnosis in the prior 3 months. Similarly, prior studies found that 93% of VHA Veteran suicide decedents diagnosed with lung cancer died by firearm and US adult suicide decedents with a history of cancer were more likely to die by firearms.^29^, ^30^ Furthermore, within US adult and Veteran suicide decedent populations, those who used firearms were less likely to have mental health diagnoses and other common suicide risk factors than those who died by other methods.^31^, ^32^ Together, this may suggest that cancer represents an acute crisis that can trigger suicidal behavior in the absence of prior mental health conditions. Alternatively, an increased propensity to firearm suicide following a cancer diagnosis may indicate that cancer's effect on suicide risk is specific to Veterans who are more likely to use a firearm method of suicide.

Strategies to prevent Veteran suicide among VHA patients diagnosed with cancer may be warranted. Root cause analyses found that a majority of VHA suicide decedents diagnosed with cancer died following a new diagnosis or change in clinical status, suggesting that these may be key intervention points for suicide prevention.^33^ Among Veterans diagnosed with cancer, post‐traumatic stress disorder (PTSD) is positively associated with suicide risk and combat‐ and cancer‐related PTSD interact to increase the likelihood of chronic pain, a well‐known suicide risk factor.^22^, ^34^, ^35^, ^36^ Additionally, factors corresponding to increased risk for suicidal‐behavior among Veterans diagnosed with head and neck cancer included prior mood disorder diagnoses and mental health and substance use disorder encounters within 90 days of cancer diagnosis. Receipt of palliative care within 90 days of diagnosis was associated with lower suicidal‐behavior risk.^37^ In addition, engaging non‐small cell lung cancer diagnosed Veterans with pre‐existing mental health conditions in mental health treatment was associated with significant decreases in all‐cause and cancer‐specific mortality.^38^ These findings indicate that suicide prevention initiatives within mental health specialties and referral to palliative care may support Veteran oncology patients.

However, awareness of oncology patient's suicide risk is necessary across all specialties. A root cause analysis among VHA oncology patients found that deaths by suicide were preceded by a lack of coordinated care, patient or staff education, or communication.^39^ These findings correspond well with several promising practices for suicide prevention in oncology settings including (1) routine distress and suicidal risk screening^40^, ^41^ (2) normalized discussion about suicidal behavior between oncologist and patients,^40^, ^42^ and (3) interdisciplinary communication and collaboration between oncologists, social workers, mental health care providers, and chaplains.^18^, ^42^, ^43^ In addition, documentation of Veteran distress in the electronic health record can facilitate clinician awareness to the social, mental, and physical needs of their cancer patients^44^ and training oncologists on lethal means safety counseling may help promote safety among oncology patients.^42^ We found that suicide risk was highest among late‐stage cancer patients who often receive systemic, non‐curative intent treatment. It is important to consider which of these suicide prevention strategies may be best suited to supplement these treatment plans.

This study had four important strengths. To our knowledge, this is the first study of the association between cancer and suicide risk specific to Veterans engaged in VHA care. Second, study analyses adjusted for important mental health and substance use disorder confounders. This was not possible in many prior US studies of cancer and suicide, which relied on the National Cancer Institute's SEER database.^3^, ^4^, ^7^ Third, use of comprehensive National Death Index death certificate search results from the VA/DoD MDR, the gold standard for death data, enhanced suicide mortality identification. Fourth, by adjusting for multiple comparisons we limited the likelihood of false positives (type I errors), which had not been accounted for in previous studies.

We note four important study limitations. First, statistical power was limited for cancer diagnoses that are uncommon within the VHA patient population, which is predominantly older males. Second, study data were limited to measures available in the VHA electronic health record, and there may be misclassification of the exposure and incomplete assessment of potential confounders, including mental health morbidity. If, as observed here, cancer is associated with increased suicide risk, then incomplete capture of cancer diagnoses may have attenuated the observed effect of cancer on suicide. Third, due to limited computing power, adjusted analyses were completed using a data driven approach; only cancer subtypes with bivariate associations with suicide risk were assessed using the full model, which adjusted for mental health comorbidities. Finally, it was beyond the scope of this study to investigate the impact of cancer treatment type, cancer progression, and VHA services utilization on suicide risk but these may be important mediators of suicide risk that should be investigated in the future.

## 5 CONCLUSION

Study findings document a positive association between a new cancer diagnosis and suicide risk among Veterans engaged in VHA care. Cancer subtypes with increased risk for suicide included esophageal, lung, and head and neck cancer as well as cancers diagnosed at stages 3 or 4 or of an unknown stage. Veterans were at highest risk for suicide in the first 3 months following diagnosis and among suicide decedents during this period firearms were more likely to be involved. VHA oncologists should be aware of and attentive to the mental health needs of their patients. Lethal means safety counseling, routine mental health screening, normalizing discussion about suicide, improving coordination and awareness between patient teams, and engaging cancer patients in mental health treatment may facilitate suicide prevention efforts within VHA oncology.

## AUTHOR CONTRIBUTIONS

Kallisse Dent curated data, conducted formal analysis, and was responsible for writing the original draft of the manuscript. Benjamin Szymanski also provided analytic input for formal analysis. John McCarthy, Ira Katz, and Michael Kelley provided supervision. All authors contributed to conceptualization, methodology, and review, and editing of the manuscript.

## FUNDING INFORMATION

This work was conducted as part of ongoing operations in the Veterans Affairs Office of Mental Health and Suicide Prevention. The views expressed in this article are those of the authors and do not necessarily reflect the position or policy of the VA.

## CONFLICT OF INTEREST

The authors have no conflict of interest to disclose.

## ETHICS STATEMENT

All analyses were conducted as a part of VA's Office of Mental Health and Suicide Prevention operations and quality improvement activities, and institutional review board was not required.

## Supporting information


Table S1

## Data Availability

Research data are not shared. We are not authorized to provide person‐level data due to HIPPA and CDC constraints related to the VA health care and death certificate data constraints, to protect patient privacy.
