# Supplementary material for: Suicide risk following a new cancer diagnosis among Veterans in Veterans Health Administration care
Source: Cancer Med. 2022 Aug 27;12(3):3520–31. doi: 10.1002/cam4.5146 (PMC9939100; doi:10.1002/cam4.5146)
Supplement: Supplementary file 1 — Table S1 [file CAM4-12-3520-s001.docx]

**Supplemental Table 1. ICD10 Cause of Death Code Definitions for Suicide Method**

| **Method of Suicide** | **ICD10 Cause of Death Codes** |
| --- | --- |
| Any Suicide | X60-X84, X87.0, and U03 |
| Firearm | X72 - X74 |
| Poisoning | X60 - X69 |
| Suffocation/Strangulation | X70 |
| Other | X71, X75 - X84, X87.0, U03 |

**Supplemental Table 2. ICD9 and ICD10 Code Definitions for Mental Health and Substance Use Disorders**

| **Diagnosis** | **ICD9 Codes** | **ICD10 Codes** |
| --- | --- | --- |
| Serious Mental Illness^1^ | 296.0, 296.1, 296.4, 296.5, 296.6, 296.7, 296.8, 295.0, 295.1, 295.2, 295.3, 295.4, 295.6, 295.7, 295.8, 295.9, 297, 298 | F30, F31, F20, F25, F06.0, F06.2, F22, F23, F24, F28, F29, F53.1 |
| Other Mental Health Disorder^2^ | 293.84, 300.0, 300.10, 300.2, 300.3, 300.7, 301.0, 301.20, 301.22, 301.4, 301.50, 301.6, 301.7, 301.81, 301.82, 301.83, 301.9, 293.83, 296.2, 296.3, 296.9, 298.0, 300.4, 301.12, 309.0, 309.1, 311, 296.2, 296.3, 300.4, 309.81 | F06.4, F40, F41, F42, F45.20, F45.21, F45.29, 301.0, 301.20, 301.22, 301.4, 301.50, 301.6, 301.7, 301.81, 301.82, 301.83, 301.9, F06.31, F06.32, F32, F33, F34.1, F53.0, F32.0, F32.1, F32.2, F32.3, F32.4, F32.5, F32.9, F33.0, F33.1, F33.2, F33.3, F33.4, F33.9, F34.1, F43.1 |
| Tobacco Use Disorder | 305.1 | F17 |
| Other Substance Use Disorder^3^ | 291, 292, 303, 304, 305.0, 305.2, 305.3, 305.4, 305.5, 305.6, 305.7, 305.8, 305.9 | F10, F11, F12, F13, F14, F15, F16, F18, F19 |
| Suicide Attempts^4^ | E950, E951, E952, E953, E954, E955, E956, E957, E958 | T14.91  T36-65 or T71 ending in 2A, 2D, 2XA, or 2XD  X71-X83  *Excluding any of the above codes ending in “S”* |

^1^Serious Mental Illness included diagnoses of bipolar disorder, schizophrenia, and other psychoses.

^2^Other Mental Health Disorder included diagnoses of anxiety, personality, major depressive, and post-traumatic stress disorder, and depression and dysthymia.

^3^Other Substance Use Disorder included diagnoses of alcohol, cannabis, cocaine, opioid, and other substance use disorder.

^4^ICD codes were only a single component of the suicide attempt definition. Suicide attempts documented by clinicians in the Veteran Health Administration’s Suicide Prevention Application Network (SPAN) or Suicide Behavior and Overdose Report (SBOR) also contributed.
